# Knockdown of lncRNA-ATB suppresses autocrine secretion of TGF-β2 by targeting ZNF217 *via* miR-200c in keloid fibroblasts

**DOI:** 10.1038/srep24728

**Published:** 2016-04-19

**Authors:** Hua-Yu Zhu, Wen-Dong Bai, Chao Li, Zhao Zheng, Hao Guan, Jia-Qi Liu, Xue-Kang Yang, Shi-Chao Han, Jian-Xin Gao, Hong-Tao Wang, Da-Hai Hu

**Affiliations:** 1 Department of Burns and Cutaneous Surgery, Xijing Hospital, Fourth Military Medical University, Xi’an, 710032, Shaanxi, People’s Republic of China; 2 Department of Hematology, Urumqi General Hospital of Chinese People’s Liberation Army, Urumqi, 830000, Xinjiang, People’s Republic of China; 3 Center of Military Burns and Plastic Surgery, Lanzhou General Hospital of Lanzhou Military Command of Chinese PLA, Lanzhou, 730050, People’s Republic of China

## Abstract

Abnormally high activation of transforming growth factor-β (TGF-β) signaling has been demonstrated to be involved in the initiation and progression of keloids. However, the functional role of long non-coding RNA (lncRNA)-activated by TGF-β (lncRNA-ATB) in keloids has not been documented. Here we investigated the role of lncRNA-ATB in the autocrine secretion of TGF-β in keloid fibroblasts (KFs) and explored the underlying molecular mechanism. Using immunohistochemistry and quantitative RT-PCR analysis, we showed that lncRNA-ATB and ZNF217, a transcriptional activator of TGF-β, were overexpressed and miR-200c, which targets ZNF217, was under-expressed in keloid tissue and keloid fibroblasts. Through gain- and loss-of-function studies, we demonstrated that knockdown of lncRNA-ATB decreased autocrine secretion of TGF-β2 and ZNF217 expression but upregulated expression of miR-200c in KFs. Stable downregulation of ZNF217 expression decreased the autocrine secretion of TGF-β2. miR-200c was endogenously associated with lncRNA-ATB, and inhibition of miR-200c overcame the decrease in ZNF217 expression in KFs. Taken together, these findings indicate that lncRNA-ATB governs the autocrine secretion of TGF-β2 in KFs, at least in part, by downregulating the expression level of ZNF217 via miR-200c, suggesting a signaling axis consisting of lncRNA-ATB/miR-200c/ZNF217/TGF-β2. These findings may provide potential biomarkers and targets for novel diagnostic and therapeutic approaches for keloids.

Keloids are benign skin tumors characterized by histological accumulation of fibroblasts and excessive deposition of extracellular matrix (ECM) components that arise as a consequence of abnormal wound healing^1^,^2^. Although it is currently known that aberrant wound healing may be mediated in part by deranged activity of growth factors^3^, including the multifunctional cytokine transforming growth factor-β (TGF-β), the mechanisms underlying keloid formation are still poorly understood^4^. Elucidating the molecular mechanisms responsible for keloid formation may promote the development of effective molecule-targeted therapies for keloids and improve the overall prognosis.

TGF-β is secreted by multiple cell types, including fibroblasts, and several isoforms exist. TGF-β isoforms belong to a superfamily of proteins involved in cellular growth and differentiation, angiogenesis, adhesion, chemotaxis, and ECM production^5^,^6^ TGF-β is known to orchestrate an intricate signaling network to modulate tumor genesis and progression^7^,^8^. Overproduction of TGF-β1 and -β2 has been associated with scar formation^9^, lung fibrosis^10^, scleroderma^11^, and other fibrotic disorders^12^. The recent literature regarding cytokine manipulation of proliferative scars has shown that TGF-β2 may be involved in the development of tissue fibrosis^13^. The synthesis of matrix proteins such as collagen, proteoglycans, and fibronectin is enhanced by TGF-β2^14^, and TGF-β1 and -β2 are thought to have profibrotic properties^15^. The role that TGF-β plays in tumors and various fibrotic diseases prompted investigation of this growth factor in the pathogenesis of keloids^5^. Abnormally high activation of TGF-β signaling has been shown to be required for the initiation and progression of keloids. These findings necessitate a better understanding of the special downstream effectors of TGF-β and a search for specific inhibitors of different TGF-β–dependent pathways for keloid treatment.

Long noncoding RNAs (lncRNAs) are a class of transcripts longer than 200 nucleotides with limited protein coding potential^16^. Studies have shown that lncRNAs play an important role in the development, growth, and progression of human carcinomas, acting as oncogenic drivers through diverse mechanisms^17^,^18^. Recently, Yuan *et al*.^7^ discovered that the lncRNA activated by TGF-β (lncRNA-ATB), an lncRNA located on chromosome 14 (ENST00000493038, http://genome.ucsc.edu/), promotes tumor cell invasion and mediates distant metastasis, exhibiting oncogenic functions. Given the fundamental biological processes regulated by TGF-β signaling and the knowledge that many of these processes are altered in keloids, we surmised that lncRNA-ATB may be involved in the pathogenesis of this benign tumor. However, until now, the functional role of lncRNA-ATB in keloids has not been documented.

MicroRNAs (miRNAs) have emerged as critical regulators of cell signaling including TGF-β signaling^19^. A previous study demonstrated that lncRNA-ATB is physically associated with the miR-200 family^7^, of which miR-200c is a well-known tumor-suppressive miRNA that is frequently downregulated in multiple cancers^20^,^21^. In addition, miR-200c showed significant downregulation in keloid tissue compared to matched normal skin tissue, suggesting that loss of miR-200c may have a role in the pathogenesis of keloids^22^. Interestingly, our previous work demonstrated that ZNF217, a transcriptional activator of TGF-β, is a target of miR-200c in breast cancer cells^8^ and promotes epithelial-to-mesenchymal transition (EMT) in breast cancer^23^. Given that TGF-β signaling is elevated in keloids, we hypothesized that lncRNA-ATB might suppress the autocrine secretion of TGF-β2 by targeting ZNF217 via miR-200c in keloid fibroblasts.

To test this hypothesis, in the present study, we detected the expression of lncRNA-ATB, miR-200c, and ZNF217 in keloid tissue and keloid fibroblasts. Through gain- and loss-of-function studies, we examined the effect of lncRNA-ATB on autocrine secretion of TGF-β2 in keloid fibroblasts as well as the interactions among TGF-β2, lncRNA-ATB, miR-200c, and ZNF217. The findings of the present study may provide potential biomarkers and targets for novel diagnostic and therapeutic approaches for keloids.

## Results

### Downregulation of lncRNA-ATB suppresses autocrine secretion of TGF-β2 in keloid fibroblasts

**Figure 1:**
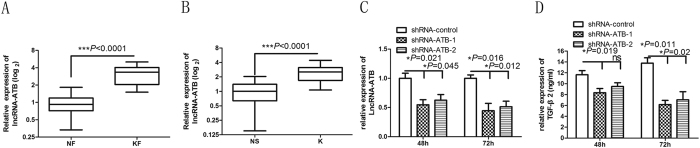
Stable knockdown of lncRNA-ATB inhibits autocrine secretion of TGF-β2 in keloid fibroblast. (**A**,**B**) qRT-PCR analysis of lncRNA-ATB levels in keloid (K) and normal skin (NS) tissues (57 paired samples), as well as in 17 pairs of keloid fibroblasts (KF) and normal fibroblasts (NF); (**C**) qRT-PCR determination of relative lncRNA-ATB expression levels in KF at 48 h and 72 h after knockdown of lncRNA-ATB with lncRNA-ATB–specific shRNAs (shRNA-ATB-1 and shRNA-ATB-2) compared with a negative control siRNA (shRNA-control); (**D**) ELISA of relative TGF-β2 expression levels in KF at 48 h and 72 h after knockdown of lncRNA-ATB.

Because lncRNA-ATB has been demonstrated to play an important role in TGF-β signaling in hepatocellular carcinoma^7^, we investigated whether lncRNA-ATB also acts similarly in keloids and keloid fibroblasts, which play a critical role in the progression of keloids. As anticipated, qRT-PCR analysis showed that lncRNA-ATB expression was significantly increased in both keloid tissue and keloid fibroblasts compared to normal skin tissue and normal fibroblasts, respectively (Fig. 1A,B).

To identify lncRNAs that are regulated by TGF-β, we treated keloid fibroblasts continuously with TGF-β1 and -β2. The results of qRT-PCR showed that the lncRNA-ATB expression was increased (Figs S1A,B), which is consistent with the data obtained from hepatocellular carcinoma cell lines^7^. Because TGF-β had a profound impact on lncRNA-ATB expression, we wonder whether lncRNA-ATB has a biological effect on TGF-β signaling. We thus used a lentivirus-mediated delivery system to stably knock down the expression of lncRNA-ATB in keloid fibroblasts. As shown in Fig. 1C,D, qRT-PCR assays and enzyme-linked immunosorbent assays (ELISAs) revealed that the level of lncRNA-ATB and TGF-β2 were significantly decreased in keloid fibroblasts. To quantify the autocrine secretion of TGF-β2 into the supernatant of keloid fibroblasts, an ELISA was performed 48 and 72 h after downregulation of lncRNA-ATB. Abrogation of lncRNA-ATB showed that the TGF-β2 downregulation was more pronounced at 72 h after transfection (Fig. 1D). This result implies that downregulation of lncRNA-ATB suppresses the autocrine secretion of TGF-β2 in keloid fibroblasts.

### ZNF217 is overexpressed in keloid tissue

**Figure 2:**
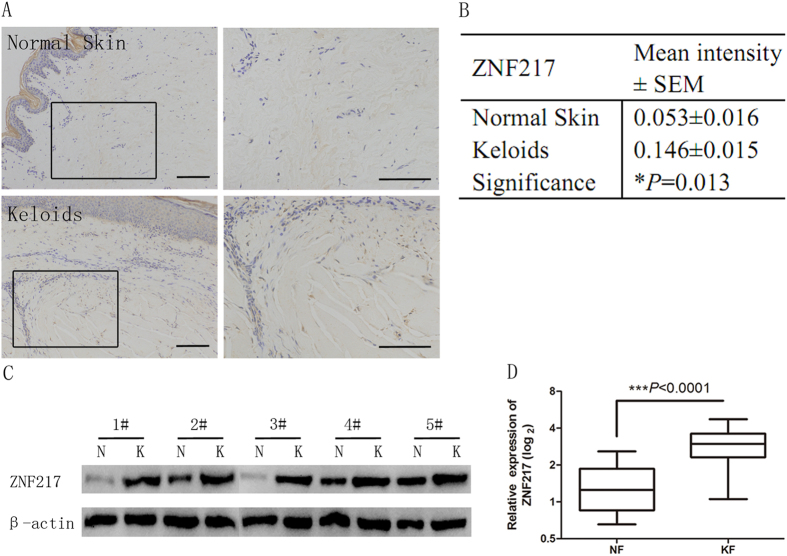
ZNF217 expression in keloid tissue and keloid fibroblasts. (**A**) Immunohistochemical staining for ZNF217 in keloid (K) tissues and paired normal skin (NS) tissues. Upper, NS; lower, K; left, ×40; right, ×200; (**B**) Comparison between K and NS tissues regarding ZNF217 expression in terms of mean intensity; (**C**) Western blot analysis of ZNF217 protein expression in normal fibroblasts (NF) and keloid fibroblasts (KF), with β-actin being used as an internal control; (**D**) qRT-PCR determination of relative expression levels of ZNF217 in NF and KF.

Given that ZNF217, a transcriptional activator of TGF-β, promotes EMT in breast cancer^8^ and that TGF-β signaling is elevated in keloids, we surmised that ZNF217 might modulate the autocrine secretion of TGF-β2 in keloids. We first determined the expression of ZNF217 protein by immunohistochemistry in keloid tissue samples from our retrospective cohort of 57 patients with keloids, whose clinicopathological data are presented in Supplemental Table 1. By signal intensity analysis, we found that ZNF217 expression was significantly increased in keloid tissue compared with normal skin tissue (*P* = 0.013; Fig. 2A,B). To confirm this finding, we next evaluated ZNF217 protein and mRNA expression in keloid tissue and normal skin tissue by Western blotting and qRT-PCR, respectively. The results showed that ZNF217 was overexpressed in keloid fibroblasts at both the protein and mRNA levels, compared to levels in normal skin tissue (Fig. 2C,D). Altogether, these data suggest that ZNF217 is overexpressed in keloid tissue and fibroblasts.

### Stable downregulation of ZNF217 expression decreases autocrine secretion of TGF-β2 *in vitro*

**Figure 3:**
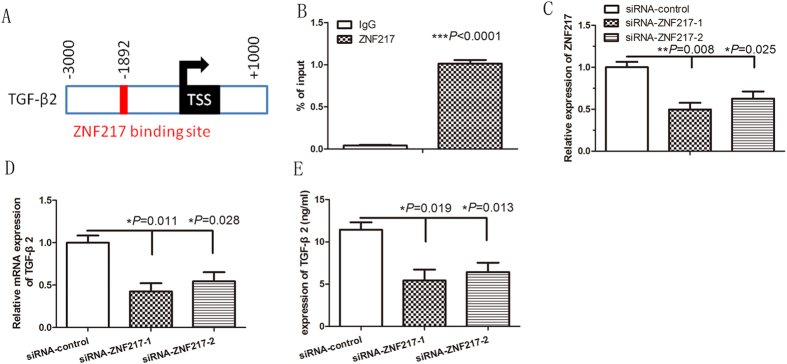
ZNF217 is a transcriptional activator of TGF-β2 in keloid fibroblasts. (**A**) Schematic representation of ZNF217 binding site in the TGF-β2 promoter region. TSS: transcription start site; (**B**) ChIP assays using anti-ZNF217 antibody or human IgG as a control on the TGF-β2 promoter region, followed by PCR using ZNF217 binding site-specific primers for measurement of TGF-β2 expression; (**C**) qRT-PCR determination of relative ZNF217 expression levels in keloid fibroblasts (KF) treated with a control siRNA (siRNA-control) or ZNF217 specific siRNAs (siRNA-ZNF217-1 and siRNA- ZNF217-2); (**D**,**E**) qRT-PCR and ELISA determination of relative TGF-β2 mRNA and protein expression levels in KF treated with ZNF217-specific siRNAs, respectively.

To determine if TGF-β2 is regulated by ZNF217, we carried out chromatin immunoprecipitation (ChIP) experiments based on several ZNF217-binding sites^23^ found in the +3 kb to −1 kb promoter region of the TGF-β2 gene (Fig. 3A). Using ChIP assays followed by PCR with primers specific to the ZNF217 proximal promoter, we found that ZNF217 could associate with the TGF-β promoter and directly upregulate the transcription of TGF-β2 (Fig. 3B).

To further investigate whether the autocrine secretion of TGF-β2 is regulated by ZNF217, we used a ZNF217 siRNA delivery system to stably transfect keloid fibroblasts. The transfection of ZNF217 siRNA decreased the expression of ZNF217 by 1.8-fold change compared with control lentivirus-infected keloid fibroblasts (Fig. 3C). ZNF217 knockdown significantly decreased TGF-β2 mRNA and protein expression (Fig. 3D,E). Collectively, these findings suggest that downregulation of ZNF217 may decrease the autocrine section of TGF-β2 in keloid fibroblasts.

### lncRNA-ATB regulates ZNF217 expression in keloid fibroblasts

**Figure 4:**
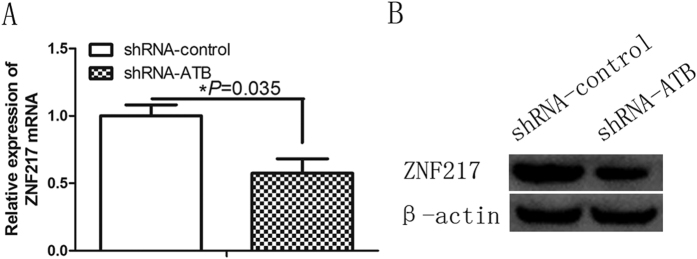
lncRNA-ATB knockdown downregulates ZNF217 expression in keloid fibroblasts. (**A**,**B**) qRT-PCR and Western blot determination of relative ZNF217 expression levels in keloid fibroblasts (KF) treated with a control shRNA (shRNA-control) or an lncRNA-ATB–specific shRNA (shRNA-ATB), respectively.

Because lncRNA-ATB and TGF-β2 reciprocally regulate each other in keloid fibroblasts, we wondered whether lncRNA-ATB could modulate ZNF217 expression. We stably knocked down lncRNA-ATB and found that the depletion of lncRNA-ATB decreased ZNF217 expression at both the mRNA and protein levels (Fig. 4A,B). These results indicate that downregulation of lncRNA-ATB inhibits ZNF217 expression in keloid fibroblasts.

### lncRNA-ATB is physically associated with miR-200c

**Figure 5:**
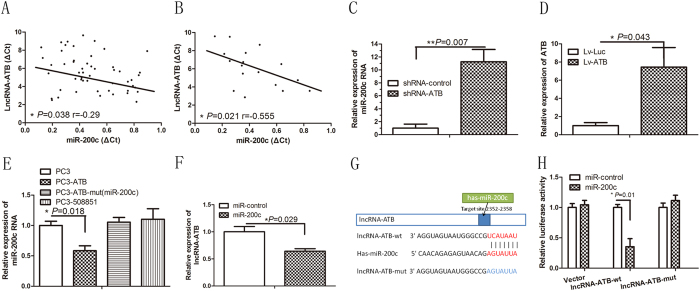
Knockdown of lncRNA-ATB upregulates miR-200c expression in keloid fibroblasts. (**A**,**B**) Pearson’s correlation analysis of relative expression levels of lncRNA-ATB and those of miR-200c determined using qRT-PCR in keloid (K) tissue and keloid fibroblasts (KF) compared to levels normal skin (NS) tissue and normal fibroblasts (NF), respectively; (**C**) quantitative RT-PCR analysis of endogenous miR-200c in KF with lncRNA-ATB knockdown using an lncRNA-ATB specific shRNA (shRNA-ATB) compared to a control shRNA (shRNA-control); (**D**) relative expression levels of lncRNA-ATB in KF overexpressing lncRNA-ATB (Lv-ATB), Lv-Luc was used as an control; (**E**) relative expression levels of miR-200c in KF transfected with an empty vector (PC3), a vector carrying wild-type lncRNA-ATB (PC3-ATB), a vector carrying mutated lncRNA-ATB [PC3-ATB-mut(miR-200c)], or a vector carrying another lncRNA induced by TGF-β and carrying none miR-200c binding site (PC3-508851); (**F**) relative expression levels of lncRNA-ATB in KF overexpressing miR-200c compared with those overexpressing a control miRNA (miR-control) is measured at 96 h; (**G**) schematic representation of the predicted binding site of miR-200c on lncRNA-ATB transcript. The nucleotides in red are the seed sequences of miR-200c; (**H**) luciferase activity in KF cotransfected with miR-200c and luciferase reporters containing nothing (vector), lncRNA-ATB (lncRNA-ATB-wt), or mutant transcript (lncRNA-ATB-mut). Data are presented as the relative ratio of firefly luciferase activity to renilla luciferase activity.

Because previous studies suggested that miR-200c may have a role in the pathogenesis of keloids^7^,^19^,^20^,^21^,^22^, we examined whether lncRNA-ATB is coexpressed with miR-200c in keloid tissue and keloid fibroblasts by measuring the expression levels of lncRNA-ATB and miR-200c by qRT-PCR. As shown in Fig. 5A,B, lncRNA-ATB transcript levels were significantly inversely correlated with miR-200c RNA levels in both keloid tissue and keloid fibroblasts.

Consistently, we found that the miR-200c RNA level was significantly upregulated in keloid fibroblasts with lncRNA-ATB knockdown (Fig. 5C). Ectopically expressed wild-type lncRNA-ATB reduced the levels of miR-200c (Fig. 5D,E). To validate whether there is endogenous direct binding between miR-200c and lncRNA-ATB, we constructed plasmids containing the 500 nt of 3′ lncRNA-ATB (PC3-ATB), lncRNA-ATB with mutations in miR-200c targeting sites (named PC3-ATB-mut), or another lncRNA - ENST00000508851 (RP11-893F2.9; named PC3-508851), which is also induced by TGF-β but does not have a predicted miR-200c targeting site (Fig. 5E). qRT-PCR analysis revealed significantly reduced expression of miR-200c in keloid fibroblasts with lncRNA-ATB (PC3-ATB) overexpression, but not in fibroblasts with PC3-ATB-mut or PC3-508851 overexpression (Fig. 5E). In addition, we found a decreased level of lncRNA-ATB after overexpression of miR-200c (Fig. 5F). Moreover, we measured the expression level of TGF-β2 in lncRNA-ATB–overexpressing keloid fibroblasts. As shown in Fig. S2, the TGF-β2 protein level was significantly increased with lncRNA-ATB up regulation. These data demonstrated that lncRNA-ATB and miR-200c can probably regulate TGF-β pathway.

To further validate the endogenous direct binding between miR-200c and lncRNA-ATB, we constructed luciferase reporters containing the 500-nt 3′ lncRNA-ATB, which includes wild-type (wt) or mutated miR-200c binding sites (Fig. 5G). We found that overexpression of miR-200c reduced the luciferase activities of the WT reporter vector but not empty vector or mutant reporter vector in keloid fibroblasts (Fig. 5H). Taken together, these data suggest that miR-200c may function as a downstream effector of lncRNA-ATB in keloid fibroblasts.

### Upregulation of miR-200c accounts for ZNF217 downregulation by lncRNA-ATB knockdown

**Figure 6:**
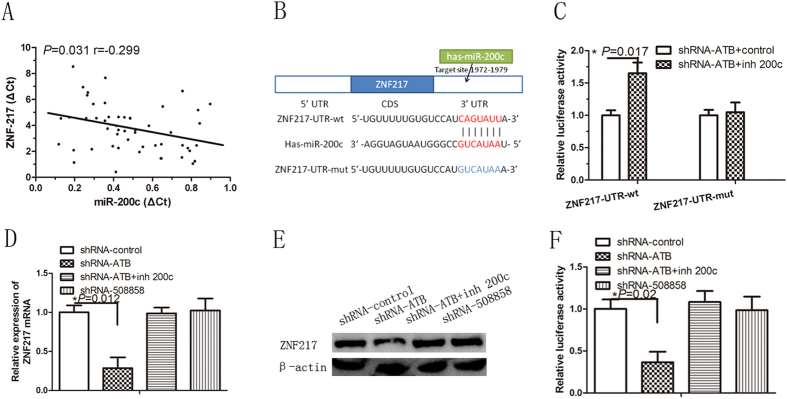
Knockdown of lncRNA-ATB upregulates miR-200c expression in keloid fibroblasts. (**A**) Correlation between miR-200c and ZNF217 mRNA expression in keloid tissue; (**B**) the predicted binding sequences of human miR-200c to the wild-type and mutant 3′-UTR of ZNF217; (**C**) relative luciferase activity in lncRNA-ATB-knockdown keloid fibroblasts (KF) co-transfected with plasmid containing wild-type (ZNF217-UTR-wt) or mutant 3′-UTR of ZNF217 (ZNF217-UTR-mut) and scrambled (control) or miR-200c inhibitor (inh 200c). Luciferase activity was measured 48 h after transfection; (**D**,**E**) the mRNA and protein expression of ZNF217 in stable KFs transfected with different shRNAs as assessed in the presence or absence of an miR-200c inhibitor (inh 200c); (**F**) luciferase activity in stable KFs transfected with luciferase reporters containing ZNF217 3′-UTR, inhibitor, or control. Data are presented as the relative ratio of firefly luciferase activity to renilla luciferase activity.

Consistent with our previous finding that ZNF217 is a target of miR-200c in breast cancer cells^8^, here we found that the mRNA levels of ZNF217 were inversely correlated with miR-200c levels in keloid fibroblasts (Fig. 6A).

To further validate the silencing of ZNF217 by miR-200c, we performed a dual luciferase reporter gene assay. The 3′-untranslated region (UTR) fragments of ZNF217, containing the potential miR-200c binding site and mutations in their seed sequences, were cloned into a vector with the firefly luciferase reporter gene (Fig. 6B). Then, these constructs were co-transfected with an miR-200c inhibitor or a control miRNA into keloid fibroblasts with lncRNA-ATB knockdown. As expected, the miR-200c inhibitor dramatically increased the expression of luciferase flanked by the 3′-UTR of ZNF217, whereas the mutations in the predicted miR-200c binding sites of 3′-UTR of ZNF217 abrogated the effect of miR-200c suppression (Fig. 6C). These results show that ZNF217 is a direct target of miR-200c in keloid fibroblasts with lncRNA-ATB knockdown.

**Figure 7:**
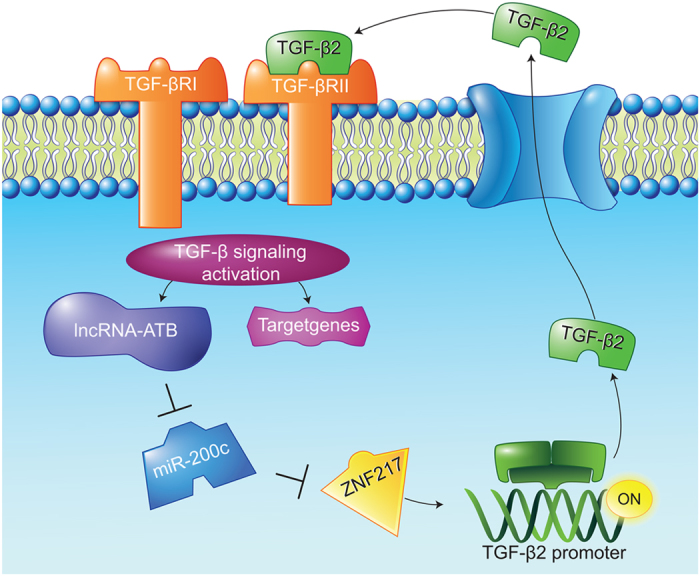
A schematic model of lncRNA-ATB functions in the pathogenesis of keloids. TGF-β signaling activation regulates a variety of downstream genes including lncRNA-ATB, which sequesters miR-200c away from mRNA of ZNF217, leading to upregulaion of ZNF217 protein and TGF-β2 transcription. Then TGF-β2 binds to TGF-βR and actives TGF-β signaling pathway reversely and forms the LncRNA-ATB/miR-200c/ZNF217/TGF-β2 loop mediating skin fibrosis.

In addition, we found that the depletion of lncRNA-ATB, but not lncRNA-508851, decreased ZNF217 expression. Inhibition of miR-200c overcame the decrease in ZNF217 expression (Fig. 6D,E). To ascertain whether this observed effect depends on regulation of the 3′-UTR of ZNF217 by miR-200c, we constructed a luciferase reporter containing the 3′-UTR of ZNF217 (pmirGLO-ZNF217) and transfected it into keloid fibroblasts. The depletion of lncRNA-ATB, but not lncRNA-508851, decreased the luciferase activity of pmirGLO-ZNF217, which was rescued by inhibition of miR-200c (Fig. 6F). All these results suggest that lncRNA-ATB promotes the autocrine secretion of TGF-β2 in keloid fibroblasts via a signaling loop involving lncRNA-ATB/miR-200c/ZNF217/TGF-β2 (Fig. 7).

## Discussion

In this study, we showed that lncRNA-ATB and ZNF217, a transcriptional activator of TGF-β, were overexpressed and miR-200c, which targets ZNF217, was under-expressed in keloid fibroblasts. We discovered that knockdown of lncRNA-ATB inhibited the autocrine production of TGF-β2 in keloid fibroblasts. We also found that miR-200c is endogenously associated with lncRNA-ATB and identified ZNF217, as a bona fide functional target of miR-200c that could be upregulated by lncRNA-ATB knockdown. Collectively, our study provides a possible novel signaling axis involving lncRNA-ATB/miR-200c/ZNF217/TGF-β2 in keloid fibroblasts.

TGF-β1 and -β2 act as fibrosis activators and mitogens for fibroblasts and can stimulate ECM deposition^24^. They regulate ECM deposition at different levels, including the promotion of ECM protein expression, the inhibition of the expression of proteases capable of degrading the ECM, the stimulation of the expression of protease inhibitors, and the regulation of integrin expression and of molecules that act as receptors for several ECM components^25^. Excessive scarring in keloid formation secondary to excessive production of ECM is the characteristic of fibrotic disease. Intriguingly, recent studies have shown that TGF-β signaling can collaborate with other molecules to regulate target genes and lncRNAs^7^,^26^. In a mouse breast cancer cell line, lncRNA-Smad7 induced by TGF-β regulates the anti-apoptotic and tumor-progressive phenotypes^26^. In multiple myeloma cells, lncRNA MALAT1 transcript activates LTBP3-regulated molecules in tumor suppressor and oncogenic pathways^27^. However, the molecular events by which TGF-β regulates or is regulated by lncRNAs in the pathogenesis of keloids, which is known to be effected by TGF-β signaling, are largely unknown.

Compared to small non-coding RNAs, lncRNAs are less well understood. Nonetheless, the few lncRNAs that have been functionally characterized are linked to diseases such as cancer, signifying that lncRNAs may not be merely transcriptional “noise”^28^. Yuan *et al*. reported that lncRNA-ATB acts not only as an endogenous miRNA sponge but also as an mRNA binder as part of its competing endogenous RNA (ceRNA) activity^7^. While we were preparing this manuscript, Iguchi *et al*. reported that the levels of lncRNA-ATB expression were significantly higher in patients with hematogenous metastases, suggesting that lncRNA-ATB may be involved in the progression of colorectal cancer and be a novel indicator of poor prognosis in patients with this malignancy^18^. Here, in our study, the finding that lncRNA-ATB is overexpressed in keloids suggests its potential utility as a therapeutic target. Soluble antisense oligonucleotides against lncRNA-ATB or other agents that block the interactions of lncRNA-ATB with target miRNAs and mRNAs may be developed to specifically block the prometastatic branch of TGF-β signaling^29^. Due to its sequence homology with mRNAs, lncRNA-ATB can sequester miR-200c like a molecular “sponge” or as a ceRNA and thereby annul miRNA function. Consistent with this, we found that knockdown of lncRNA-ATB remarkably increased endogenous miR-200c levels in keloid fibroblasts. Identifying the functional role of lncRNAs and relative miRNAs aberrantly expressed in tumor tissues as well as the underlying mechanism would provide a novel avenue for the exploration of miRNA-based diagnostics and therapeutics.

The elucidation of gene-regulatory networks that control gene expression programs represents a major challenge in cell biology^30^. Subsequent to the discovery of miRNAs, multiple lines of evidence demonstrated that miRNA-associated networks play a critical role in disease initiation and progression^30^,^31^. In addition, recent studies demonstrated that TGF-β signaling can be targeted by different miRNAs^32^,^33^, strongly suggesting that expression and function of TGF-β family members are under rigorous surveillance of tumor-suppressive miRNAs in normal fibroblasts. miR-200c, belonging to the miRNA-200 family, might play an oncogenic role in non-small cell lung cancer^34^, but might be a tumor suppressive miRNA in breast cancer cell lines^35^. The role of miR-200c in the skin has not been firmly established^36^. Confirming the role of miRNA-200c in keloids may improve the understanding of the pathogenesis of cutaneous fibrotic diseases^36^. In this study, we found that miR-200c, by targeting ZNF217, regulates the autocrine secretion of TGF-β, thus creating a signaling axis involving lncRNA-ATB, miR-200c, ZNF217, and TGF-β2. During the process of keloid progression, the oncogenic lncRNA-ATB is activated and contributes to the imbalance between miR-200c and ZNF217, resulting in abnormal TGF-β expression. As an important tumor-suppressive miRNA, miR-200c regulates a large cohort of oncogenes and functions as the master regulator of multiple pathways. Thus, the process of keloid progression would be, at least in part, accelerated upon the loss of miR-200c.

## Conclusions

Our results provide the first evidence that in keloids, lncRNA-ATB governs autocrine secretion of TGF-β2, at least in part, by downregulating the expression level of ZNF217 via miR-200c. Our findings, together with those of previous studies, indicate that miR-200c is part of a regulatory pathway that inhibits autocrine production of TGF-β2. Thus, both lncRNA-ATB and miR-200c might be potential biomarkers and targets for novel diagnostic and therapeutic approaches for keloids.

## Materials and Methods

### Tissue samples

Keloid tissueand paired normal skin tissue samples were surgically obtained from 57 patients who were diagnosed pathologically with keloids at the Department of Burns and Cutaneous Surgery of Xijing Hospital between April 2009 and July 2014. All the protocols were approved by the Ethics Committee of Xijing Hospital affiliated to Fourth Military Medical University (China). Written informed consent was obtained for each participant according to the guidelines. The collection of skin samples was carried out in accordance with the approved guidelines. The collected skin samples were divided into three portions; one was fixed in 4% paraformaldehyde for histopathological examination, the second was soaked in liquid nitrogen for the preparation of total RNA and total protein lysates, and the third was used for the isolation and culture of fibroblasts.

### Cell culture

Cultures of keloid fibroblasts and normal skin fibroblasts, which were isolated from 17 pairs of keloid tissue samples and normal skin samples, respectively, were established as described previously^1^,^3^. All cells were maintained in a humidified incubator at 37 °C in an atmosphere containing 5% CO_2_. Fibroblasts at the third to fifth passages were used in all experiments unless otherwise indicated.

### Quantitative real-time PCR analysis

RNA extraction and real-time PCR were performed as previously described^8^ using the following primers: lncRNA-ATB reverse transcription: 5′-ACA CAG AAT AAA ATA ACA C-3′; lncRNA-ATB forward: 5′-TCT GGC TGA GGC TGG TTG AC-3′, reverse: 5′-ATC TCT GGG TGC TGG TGA AGG-3′; lncRNA-508851 forward: 5′-GAT CAA AGT TCA CAA GGC A-3′, reverse: 5′-GAG TGG GAT GTA GGT AGC-3′; ZNF217 forward: 5′-AAA CAT GCC AAC TCA ATC CCT C-3′, reverse: 5′-GGA ATG GAA CAA CAG CGG T-3′; GAPDH (as an endogenous control) forward: 5′-TCA CCA GGG CTG CTT TTA AC-3′, reverse: 5′-GAC AAG CTT CCC GTT CTC AG-3′; miR-200c forward: 5′-UAA UAC UGC CGG GUA AUG AUG GA-3′, reverse: Universal Primer (QIAGEN, Germany); and U6-snRNA forward: RNU6B_2 miScript Primer (QIAGEN), reverse: Universal Primer (QIAGEN).

### miRNA mimics and siRNA transfection

All synthetic miRNAs including negative control (miR-control) and miR-200c were purchased from Shanghai Genechem (Shanghai, China). Silencer Select Negative Control siRNAs (siRNA control), siRNA-ZNF217-1 (5′-CGA UCA ACG AGG UCG UCC A-3′), and siRNA-ZNF217-2 (5′-ACU GCU UUC GGU ACC AGC A-3′) were obtained from Genechem.

### ELISA

Cell culture supernatants were collected in sterile tubes and stored at −20 °C until use. The concentration of TGF-β2 was determined using an ELISA kit (R&D Systems, Wiesbaden, Germany) according to the manufacturer’s instructions^37^. All analyses and calibrations were carried out in duplicate. All concentrations were recorded as ng/mL. To quantitate the autocrine secretion of TGF-β2, an ELISA was performed 48 and 72 h after lncRNA-ATB knockdown.

### Immunohistochemical staining, microscopy, and image analysis

Immunohistochemical staining, microscopy, and image analysis were performed as previously described^38^,^39^,^40^. The primary antibody used was anti-ZNF217 rabbit polyclonal antibody (1:200; ab181741, Abcam, Cambridge, UK), and goat anti-rabbit IgG (Alexa Fluor 594, Invitrogen) was used as a secondary antibody. The signal intensity of tissue areas from five randomly selected views was blindly determined and statistically analyzed.

### Western blot analysis

Western blot analysis was performed as previously described^8^. The primary antibodies used included anti-ZNF217 rabbit polyclonal antibody (1:500; ab181741, Abcam) and anti-β-actin mouse monoclonal antibody (1:1000; A5441, Sigma-Aldrich, St. Louis, MO, USA). Goat anti-mouse IgG (Alexa Fluor 488, Invitrogen) and goat anti-rabbit IgG (Alexa Fluor 594, Invitrogen) were used as secondary antibodies.

### ChIP

ChIP experiments were performed as previously described^7^. Briefly, keloid fibroblasts were cultured, washed twice with phosphate-buffered saline (PBS), and cross-linked using 1% formaldehyde for 10 min. Following lysis of the cells and sonication, DNA–protein complexes in the lysates were subjected to immunoprecipitation using anti-ZNF217 (ab117798, Abcam) or control normal IgG. After precipitation of the immunocomplex with protein G-agarose, isolated DNA was used as template in PCR with specific primers spanning the target region of the TGF-β2 promoter. Primers used for amplification of the human TGF-β2 promoter were as follows: forward, 5′-CGG GAG ACT TGA TTG TCC TT-3′ and reverse, 5′-CGT TGA GGG AGT GTG GAA AT-3′.

### Vector construction, lentivirus production, and construction of stable cell lines

The cDNA encoding lncRNA-ATB or lncRNA-508851 (another lncRNA that is also induced by TGF-β but does not have a predicted miR-200c targeting site) was PCR-amplified and subcloned into pcDNA3.1 vector (Invitrogen), named PC3-ATB and PC3-508851, respectively. The PC3-ATB with point mutations in the miR-200c response elements was synthesized using a QuikChange Site-Directed Mutagenesis kit (Stratagene) and named PC3-ATB-mut (miR-200c)^7^. The 525-nt region at the 3′ end of either lncRNA-ATB or lncRNA-ATB-mut (miR-200c) was amplified using PCR and subcloned into the pmirGLO vector (Promega, Madison, WI) for a luciferase reporter assay using the one-step directed cloning kit (Novoprotein, Shanghai, China)^7^. The human miR-200c precursor sequence was amplified by PCR using the primers 5′-GAA TTC GGG GAT CTG GGC GCA GGG GCC-3′ and 5′-CTC GAG CCG ACC TGG AGC GCG GAG C-3′ and cloned into a pcDNA3.1 vector, and the resulting vector was named PC3-200c. Lentivirus production and generation of cell lines stably expressing lncRNA-ATB, lncRNA-ATB-mut (miR-200c), or lncRNA-508851 were performed as previously described^7^. For construction of lentiviral vector expressing the human lncRNA-ATB gene, lncRNA-ATB cDNA was PCR-amplified and subcloned to the lentiviral vector, named Lv-ATB, using Lv-Luc as control^7^. We used a scrambled shRNA as the negative control for shRNA-ATB-1, shRNA-ATB-2, and shRNA-508851 and designated this as shRNA-control^7^.

### Luciferase assay

The 3′-UTR fragments of ZNF217 containing the putative binding site for miR-200c and 3′UTR-mutation were modified as previously described^8^. The fragments were subcloned into pmirGLO vector, a dual-luciferase miRNA target expression vector (Promega). pmirGLO, pmirGLO-ATB, or pmirGLO-ATB-mut (miR-200c) was cotransfected with miR-200c mimic or miR NC (a negative control), mirGLO (as a control), or pmirGLO-ZNF217 into keloid fibroblasts by Lipofectamine-mediated gene transfer^7^. Luciferase assays were performed 48 h after transfection using the dual-luciferase reporter assay system. Firefly luciferase activity was normalized to renilla luciferase expression for each sample.

### Statistical analysis

All statistical analyses were performed using GraphPad Prism software (version 5.0; GraphPad Software, San Diego, CA, USA). For comparisons, Student’s *t*-tests (two-tailed) were performed as indicated. The paired t-test was used to compare the mean signal intensity between keloid and normal skin tissues.

## Additional Information

**How to cite this article**: Zhu, H.-Y. *et al*. Knockdown of lncRNA-ATB suppresses autocrine secretion of TGF-β2 by targeting ZNF217 *via* miR-200c in keloid fibroblasts. *Sci. Rep*. **6**, 24728; doi: 10.1038/srep24728 (2016).

## Supplementary Material

Supplementary Information
