# Supplementary material for: Knockdown of lncRNA-ATB suppresses autocrine secretion of TGF-β2 by targeting ZNF217 via miR-200c in keloid fibroblasts
Source: Sci Rep. 2016 Apr 19;6:24728. doi: 10.1038/srep24728 (PMC4835760; doi:10.1038/srep24728)
Supplement: Supplementary Information [file srep24728-s1.doc]

# Knockdown of lncRNA-ATB suppresses autocrine secretion of TGF-β2 by targeting ZNF217 *via* miR-200c in keloid fibroblasts

Hua-Yu Zhu^1,#^, Wen-Dong Bai^2,#^, Chao Li^1,3,#^, Zhao Zheng^1^, Hao Guan^1^, Jia-qi Liu^1^, Xue-kang Yang^1^, Shi-chao Han^1^, Jian-xin Gao^1^, Hong-tao Wang ^1,*^, Da-Hai Hu^1,*^


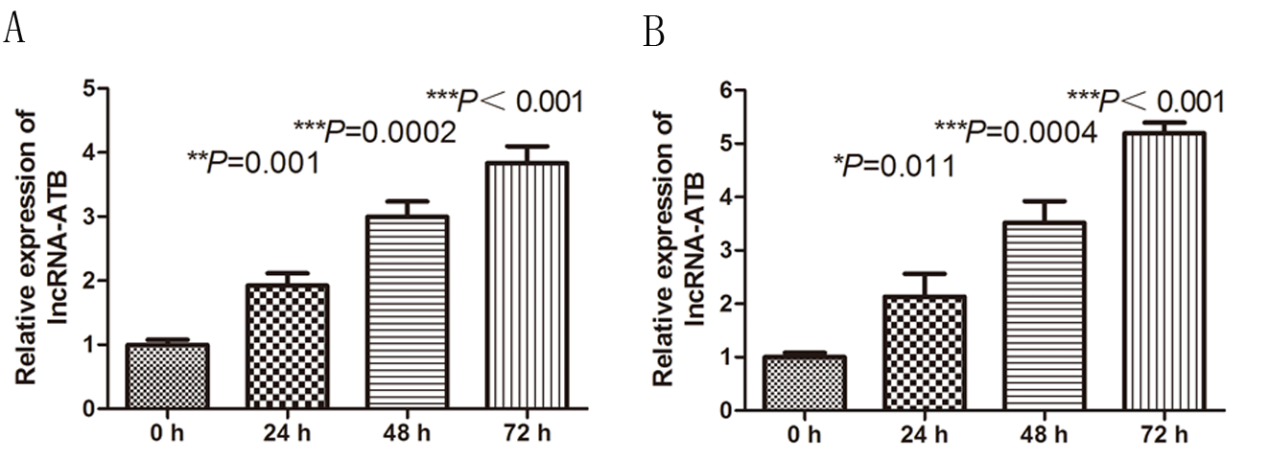


Figure S1. TGF-β–related expression of lncRNA-ATB in keloid fibroblasts. KFs were treated with 10 ng/ml of recombinant TGF-β1 (A) and TGF-β2 (B) for the indicated times as measured by qRT-PCR.


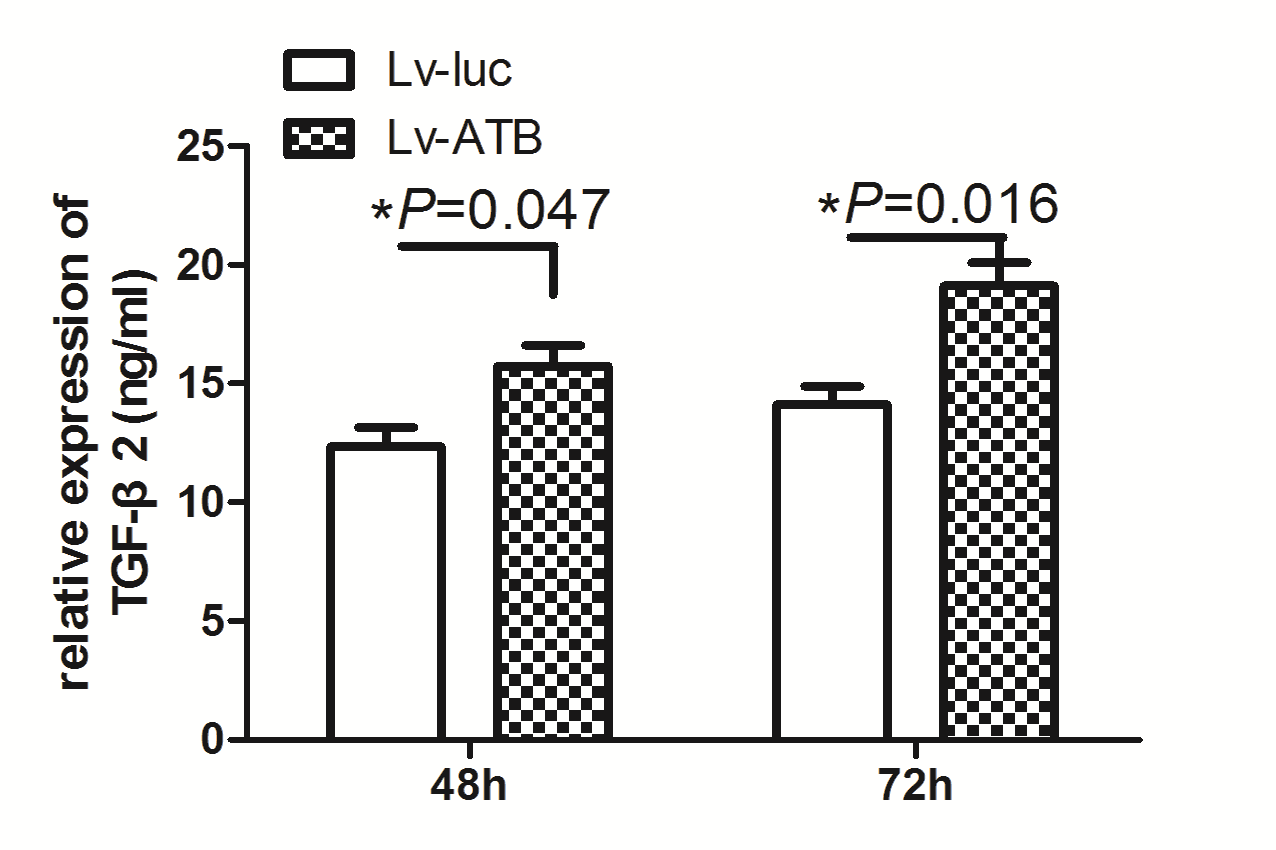


Figure S2. LncRNA-ATB upregulates TGF-β2 expression in keloid fibroblasts. ELISA for TGF-β2 expression in KFs at 48 and 72 h after overexpression of lncRNA-ATB.

| **TABLE 1.** **Clinicopathological data for keloid and normal skin tissues*** | |
| --- | --- |
|  | **Keloids/ Normal Skin* N =57** |
| **Age/years** |  |
| Mean ± SD | 31.4 ± 8.4 |
| Range | 12–54 |
| **Duration/months** |  |
| Mean ± SD | 36.2 ± 19.2 |
| Range | 7–122 |
| **Sex** |  |
| Female  Male | 29 (50.8%)  28 (49.2%) |
| **Cause of lesion** |  |
| Traumatic | 21 (36.8%) |
| Surgery | 19 (33.3%) |
| Spontaneous | 17 (29.9%) |
| **Site of lesion** |  |
| Head, face, and neck | 28 (49.1%) |
| Truncus | 16 (28.1%) |
| Extremities | 13 (22.8%) |
| **Stage of lesion** |  |
| Immature scar | 31(54.4%) |
| Mature scar | 26 (45.6%) |

*Normal skins were obtained from areas adjacent to corresponding keloid tissues.
